# Accuracy of ROSA^®^ Partial Knee System in Tibial Alignment During Medial Unicompartmental Knee Arthroplasty: An Observational Study

**DOI:** 10.3390/jcm15103566

**Published:** 2026-05-07

**Authors:** Stefano Petrillo, Damiano Ardiri, Paolo Perazzo, Sergio Romagnoli, Matteo Marullo

**Affiliations:** Prosthetic Surgery Centre, IRCCS Galeazzi Sant’Ambrogio, 20157 Milan, Italy; stefano.g.petrillo@gmail.com (S.P.); paoloperazzo1@virgilio.it (P.P.); sergio.romagnoli@libero.it (S.R.); matteomarullo@hotmail.it (M.M.)

**Keywords:** medial UKA, ROSA Partial Knee, imageless robotics, tibial alignment, MPTA, tibial slope

## Abstract

**Background/Objectives:** The ROSA^®^ Partial Knee System (Zimmer Biomet, Warsaw, IN, USA) is an imageless robotic platform developed to improve the reproducibility of tibial resection and implant positioning in unicompartmental knee arthroplasty (UKA). Evidence on imageless ROSA-assisted medial UKA remains limited. This preliminary single-center study evaluated the early radiographic accuracy of the system in reproducing planned tibial coronal alignment and tibial slope during robotic-assisted medial UKA, while perioperative and short-term clinical findings were assessed as secondary exploratory observations. **Methods:** A retrospective analysis of a prospectively maintained database was performed on 23 consecutive patients who underwent robotic-assisted medial UKA using the ROSA^®^ Partial Knee System and Persona^®^ Partial Knee implant between December 2025 and March 2026. Planned and robot-validated values were compared with postoperative radiographic measurements, which were used as the achieved alignment reference. Deviations >2° and >3° from target were defined as outliers. Paired clinical analyses were restricted to patients with data available at both time points. **Results:** Mean age was 68.4 ± 8.5 years and mean BMI was 28.2 ± 4.1 kg/m^2^. Preoperative HKA was 5.4 ± 3.4° varus, while final robot-validated and postoperative radiographic HKA values were 2.3 ± 0.7° and 2.6 ± 0.9° varus, respectively (*p* = 0.08). Robot-validated and postoperative radiographic MPTA values were 2.0 ± 0.5° and 2.1 ± 0.7°, respectively (*p* = 0.41). Planned, robot-validated, and postoperative radiographic tibial slope values were 4.5 ± 0.6°, 4.6 ± 0.6°, and 4.8 ± 0.8°, respectively (*p* = 0.27). Mean absolute postoperative deviation from target was 0.42 ± 0.38° for HKA, 0.34 ± 0.29° for MPTA, and 0.46 ± 0.39° for tibial slope. Within 2° of target, accuracy ranged from 91.3% to 95.7%, and all cases were within 3°. Short-term clinical outcomes improved in the available paired subsets (n = 14 for VAS and KSS, n = 12 for ROM, and n = 9 for UCLA activity score). **Conclusions:** In this preliminary single-center observational series, the ROSA^®^ Partial Knee System showed high early radiographic accuracy in reproducing planned tibial coronal alignment and tibial slope during robotic-assisted medial UKA, with low outlier rates. Short-term clinical findings were favorable in the available paired subsets but should be interpreted as exploratory. Larger comparative studies with longer follow-up are needed to determine the clinical relevance of this technical accuracy.

## 1. Introduction

Unicompartmental knee arthroplasty (UKA) is an established treatment option for selected patients with isolated compartmental knee osteoarthritis, providing satisfactory clinical outcomes, high implant survivorship, and preservation of more physiological knee kinematics compared with total knee arthroplasty in appropriately indicated cases [1,2,3,4,5]. Despite these advantages, UKA remains a technically demanding procedure, and its success is strongly influenced by accurate implant positioning and restoration of lower limb alignment.

Proper alignment and component positioning are crucial determinants of implant function, polyethylene wear, and long-term survivorship in UKA [6,7,8,9,10,11,12]. Coronal malalignment, excessive or insufficient tibial slope, and rotational errors may adversely affect load distribution, bearing wear, ligament balance, and implant longevity [6,7,8,9,10]. Early failures of UKA have often been associated with technical errors, suboptimal component positioning, and inappropriate restoration of joint geometry [11,12]. More recently, robotic-assisted UKA has been associated with improved implant positioning accuracy, reduced alignment outliers, and better restoration of native joint line parameters when compared with conventional techniques [13,14,15,16].

Conventional and minimally invasive UKA techniques may further increase the technical difficulty of the procedure because of reduced surgical exposure, and limited visualization [17,18,19,20]. Inaccurate implant positioning during minimally invasive surgery may compromise the theoretical advantages of UKA and may contribute to early revision, suboptimal functional outcomes, or reduced implant survival [17,18,19,20]. For these reasons, technologies aimed at improving reproducibility and reducing technical variability have progressively gained interest in knee arthroplasty.

Computer-assisted navigation was introduced to improve intraoperative control of alignment and implant positioning in UKA, with encouraging early results in terms of coronal alignment and reproducibility [21,22,23]. Building on these concepts, robotic systems have been developed to further enhance the precision of bone preparation and component implantation [24,25,26,27,28,29,30]. In recent years, growing evidence has supported the use of robotic-assisted UKA, especially with robotic-arm platforms, showing improved radiographic accuracy and fewer alignment outliers compared with conventional techniques [31,32,33,34,35,36,37,38,39,40]. However, whether improved radiographic precision consistently translates into superior clinical outcomes or survivorship remains incompletely established.

However, most currently available evidence concerns robotic-arm systems, while data on imageless robotic-assisted UKA remain limited. In particular, evidence on the radiographic accuracy of the ROSA^®^ Partial Knee System in medial unicompartmental knee arthroplasty remains limited, while short-term clinical observations are still preliminary. The present investigation primarily evaluated the radiographic accuracy of the ROSA^®^ Partial Knee System in reproducing planned tibial coronal alignment and tibial slope during robotic-assisted medial UKA. Perioperative findings and short-term clinical outcomes were assessed as secondary exploratory observations. We hypothesized that radiographic outlier rates would remain low.

## 2. Materials and Methods

The present study was conducted in accordance with the Strengthening the Reporting of Observational Studies in Epidemiology (STROBE) guidelines. The study protocol was approved by the Comitato Etico Territoriale Lombardia 1 (CET Lombardia 1) within the retrospective clinical evaluation protocol ALLCCP promoted by IRCCS Ospedale Galeazzi-Sant’Ambrogio. The study was performed in accordance with the principles of the Declaration of Helsinki and its later amendments. All patients understood the nature of their treatment and provided written informed consent for the use of their clinical and imaging data for research purposes.

A retrospective analysis of a prospectively maintained institutional database was performed on 23 consecutive patients who underwent robotic-assisted medial unicompartmental knee arthroplasty (UKA) using the ROSA^®^ Partial Knee System and the Persona^®^ Partial Knee implant (Zimmer Biomet, Warsaw, IN, USA) between December 2025 and March 2026 at the Joint Replacement Department, IRCCS Ospedale Galeazzi-Sant’Ambrogio, Milan, Italy. This investigation was designed primarily as an early radiographic accuracy study of a recently introduced imageless robotic platform. Perioperative variables and short-term clinical outcomes were assessed as secondary exploratory endpoints.

Eligibility criteria were defined before data extraction. Inclusion criteria were: adults undergoing primary robotic-assisted medial UKA for symptomatic isolated medial compartment knee osteoarthritis, failure of conservative treatment, and availability of complete preoperative, intraoperative, and postoperative radiographic data in the institutional database. Exclusion criteria were: revision procedures, active infection, incomplete robotic surgical reports, missing postoperative radiographs, and incomplete follow-up for the specific outcome of interest in paired clinical analyses.

The completed STROBE checklist is provided as Supplementary Material S1, and the study flow diagram describing patient selection is provided as Supplementary Material S2. No a priori sample size calculation was performed because this was an exploratory early technology evaluation.

### 2.1. Surgical Procedure

All procedures were performed by the senior author [S.P] using a standardized operative workflow. Robotic-assisted medial UKA was performed with the ROSA^®^ Partial Knee System and the Persona^®^ Partial Knee implant (Zimmer Biomet, Warsaw, IN, USA) through a mid-vastus approach in all cases.

After surgical exposure, femoral and tibial tracker pins were positioned according to the manufacturer’s recommendations, and anatomical registration was completed. Pre-resection planning was then performed using ROSA^®^ Partial Knee System, with intraoperative adjustment of coronal alignment and tibial slope according to the preoperative deformity, soft-tissue balance, and intended component position. The robotic workflow was subsequently used to guide bone preparation and to validate final implant positioning intraoperatively. Trial reduction and definitive implantation were performed after verification of alignment, stability, and range of motion. Postoperative rehabilitation was standardized for all patients.

### 2.2. Outcomes

The primary outcome of the present investigation was the radiographic accuracy of the robotic system in reproducing the planned tibial coronal alignment and tibial slope during robotic-assisted medial UKA. Planned intraoperative values were extracted from the robotic surgical report. Final robot-validated values were recorded intraoperatively, and postoperative radiographic measurements were considered the achieved alignment reference for comparison with planned targets. Deviations greater than 2° and 3° from the planned target were defined a priori as radiographic outliers.

Secondary radiographic outcomes included the hip–knee–ankle angle (HKA), measured preoperatively on full-length standing radiographs, as well as final robot-validated HKA values and postoperative HKA measured on full-length standing radiographs. Additional implant-related radiographic parameters included robot-validated and postoperative medial proximal tibial angle (MPTA) and tibial slope. The radiographic parameters evaluated in the study and their reporting rationale are summarized in Table 1.

Perioperative outcomes included robotic time, total surgical duration, preoperative hemoglobin, and hemoglobin drop. Robotic time was defined as the time required for the robotic portion of the procedure, including tracker placement, registration, planning, and robotic validation steps. Total surgical duration was defined as skin incision to wound closure.

Short-term clinical outcomes were assessed as secondary exploratory endpoints and included pain assessed using the visual analogue scale (VAS), Knee Society Score (KSS), University of California Los Angeles (UCLA) activity score, Forgotten Joint Score-12 (FJS-12), and range of motion (ROM), according to data availability. Because follow-up availability differed across outcome measures, paired clinical analyses were restricted to patients with data available at both baseline and 3-month follow-up for the specific variable under evaluation.

Postoperative radiographic assessment was performed under routine clinical practice conditions at the first standardized postoperative radiographic follow-up, usually around 3 months after surgery. Standardized full-length weight-bearing anteroposterior lower-limb radiographs and lateral knee radiographs were obtained according to the institutional follow-up protocol. All radiographic measurements were performed using Sectra IDS7, version 27.2 (Sectra AB, Linköping, Sweden) by a single experienced observer under routine evaluation conditions. The observer was not formally blinded to the clinical setting or to the intraoperative robotic values. To assess measurement consistency, a subset of radiographs was re-evaluated by the same observer, and intraobserver reliability was quantified using the intraclass correlation coefficient (ICC). Intraobserver reliability was excellent for HKA (ICC = 0.92), good for MPTA (ICC = 0.88), and good for tibial slope (ICC = 0.86).

### 2.3. Statistical Analysis

All statistical analyses were performed by a biomedical statistician using IBM SPSS Statistics for Windows, version 29.0 (IBM Corp., Armonk, NY, USA). Descriptive statistics were used to summarize the data and included absolute and relative frequencies for categorical variables and means with standard deviations for continuous variables. Data distribution was assessed using the Shapiro–Wilk test.

Given the exploratory nature of the study and the limited sample size, the radiographic accuracy analysis was interpreted primarily descriptively, with particular emphasis on absolute postoperative deviation from target values and outlier rates. Paired comparisons between robot-validated and postoperative radiographic values were performed using the Wilcoxon signed-rank test. The same test was used for paired comparisons between baseline and 3-month clinical outcomes in the available paired subsets. Accordingly, the number of paired observations is reported for each clinical endpoint.

No imputation of missing data was performed. Clinical analyses were restricted to available paired observations, and the paired sample size therefore differed across outcomes according to follow-up availability.

For the primary radiographic accuracy analysis, the absolute deviation between the planned target and the postoperative radiographic measurement was calculated for HKA, MPTA, and tibial slope. Outliers were defined as deviations greater than 2° and greater than 3° from the target value. Continuous data are reported as mean ± standard deviation, and statistical significance was set at *p* < 0.05.

## 3. Results

### 3.1. Patient Demographics and Perioperative Data

Twenty-three consecutive patients undergoing robotic-assisted medial UKA were included in the present investigation. All 23 patients had complete radiographic data and were therefore included in the radiographic accuracy analysis. The cohort comprised 14 men and 9 women, with a mean age of 68.4 ± 8.5 years and a mean body mass index of 28.2 ± 4.1 kg/m^2^. Kellgren–Lawrence grade 3 osteoarthritis was present in 9 knees (39.1%), while 14 knees (60.9%) had grade 4 degeneration. Mean preoperative HKA was 5.4 ± 3.4° varus. Perioperative data showed a mean robotic time of 15.8 ± 2.7 min, a mean total surgical duration of 29.4 ± 4.8 min, and a mean hemoglobin drop of 0.8 ± 0.4 g/dL (Table 2).

### 3.2. Radiographic Accuracy and Outliers

Radiographic accuracy findings are summarized in Table 3, Table 4, Table 5 and Table 6. Given the small sample, agreement between planned or robot-validated values and postoperative radiographic measurements is best interpreted descriptively through absolute deviation and outlier rates rather than through hypothesis testing alone. Mean absolute deviation from target was 0.42 ± 0.38° for HKA, 0.34 ± 0.29° for tibial coronal alignment (MPTA), and 0.46 ± 0.39° for tibial slope. Within 2° of target, the percentage of cases was 91.3% for HKA, 95.7% for MPTA, and 91.3% for tibial slope. All cases were within 3° of target for the three evaluated parameters.

**Figure 1:**
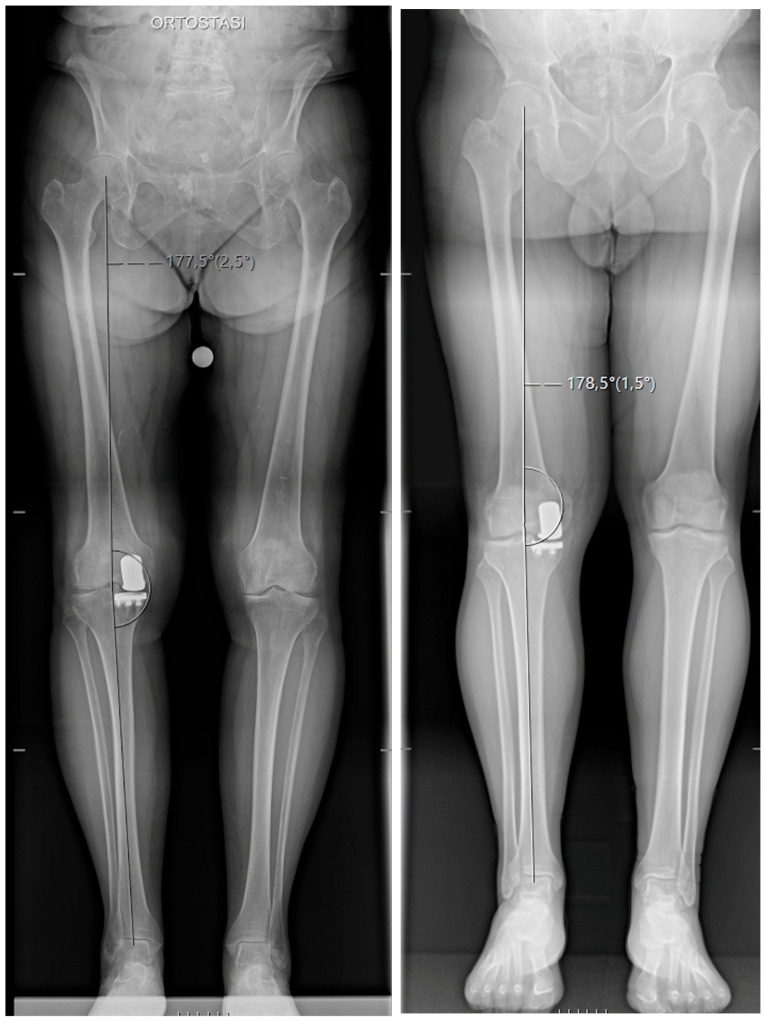
Representative full-length standing anteroposterior radiographs after robotic-assisted medial unicompartmental knee arthroplasty, illustrating postoperative coronal lower-limb alignment in relation to the radiographic accuracy endpoints assessed in the study.

Final robot-validated and postoperative radiographic coronal values were similar for both HKA (2.3 ± 0.7° varus vs. 2.6 ± 0.9° varus; *p* = 0.08) and MPTA (2.0 ± 0.5° vs. 2.1 ± 0.7°; *p* = 0.41). Representative postoperative full-length standing anteroposterior radiographs illustrating coronal alignment are shown in Figure 1.

**Figure 2:**
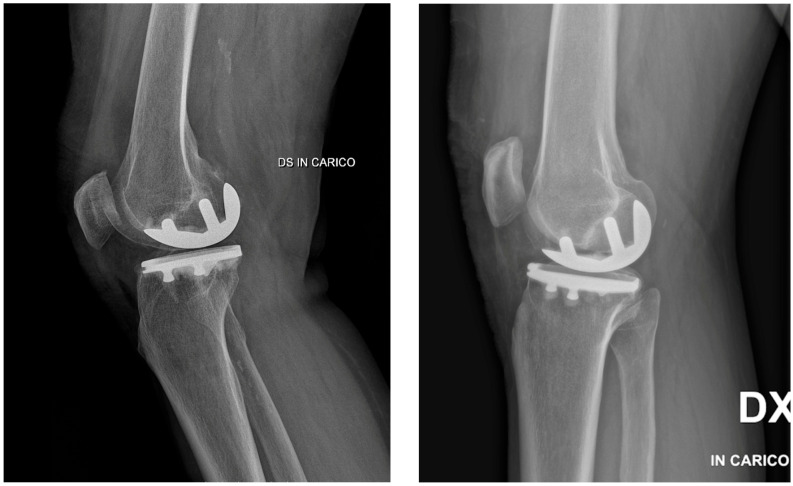
Representative lateral radiographs after robotic-assisted medial unicompartmental knee arthroplasty, illustrating postoperative sagittal implant positioning and tibial slope in relation to the radiographic accuracy endpoints assessed in the study.

Likewise, planned, robot-validated, and postoperative radiographic tibial slope values were 4.5 ± 0.6°, 4.6 ± 0.6°, and 4.8 ± 0.8°, respectively (*p* = 0.27). Representative postoperative lateral radiographs illustrating sagittal implant positioning and tibial slope are shown in Figure 2.

**Figure 3:**
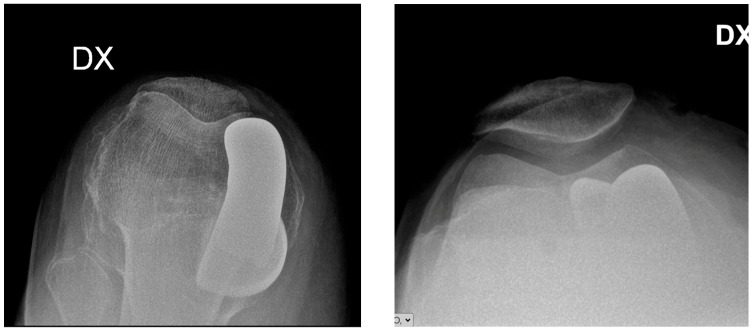
Representative axial patellar radiographs after robotic-assisted medial unicompartmental knee arthroplasty, provided as complementary postoperative imaging to illustrate patellofemoral tracking and overall implant appearance.

Complementary axial patellar radiographs documenting overall implant appearance and patellofemoral tracking are provided in Figure 3.

Intraobserver reliability was excellent for HKA and good for MPTA and tibial slope, with ICC values of 0.92, 0.88, and 0.86, respectively.

### 3.3. Early Clinical Outcomes

Short-term clinical outcomes at approximately 3 months are reported in Table 7. Because follow-up availability differed across outcome measures, paired analyses were outcome-specific and should be interpreted as preliminary. In the available paired subset, VAS improved from 8.1 ± 0.7 to 1.0 ± 0.9 (paired n = 14; *p* < 0.001), KSS Function from 39.0 ± 6.2 to 86.5 ± 7.8 (paired n = 14; *p* < 0.001), and KSS Knee from 41.2 ± 7.0 to 91.8 ± 6.5 (paired n = 14; *p* < 0.001).

ROM outcomes were available in 12 paired cases. Extension deficit decreased from 2.1 ± 3.4° to 0.2 ± 0.8° (paired n = 12; *p* = 0.04), while flexion increased from 121.5 ± 8.8° to 129.4 ± 6.7° (paired n = 12; *p* = 0.01). UCLA activity score improved from 3.2 ± 0.6 to 5.6 ± 1.0 in the paired subset with available data (paired n = 9; *p* = 0.008).

Postoperative FJS-12 data were not sufficiently mature for formal inferential analysis and were therefore not included in the paired statistical results.

## 4. Discussion

The main finding of the present investigation is that robotic-assisted medial UKA performed with the ROSA^®^ Partial Knee System showed high early radiographic accuracy in reproducing planned tibial coronal alignment and tibial slope in this preliminary single-center series. The most informative findings were the low mean absolute deviations from target for HKA, MPTA, and tibial slope, the high proportion of cases within 2° of target, and the absence of >3° outliers for all three evaluated parameters. Perioperative findings were also favorable. Short-term clinical observations were encouraging in the available paired subsets, although they should be interpreted as secondary exploratory findings because follow-up availability differed across outcomes.

Component positioning and restoration of limb alignment are crucial in UKA, since coronal malalignment, rotational errors, and inappropriate posterior slope may affect load distribution, polyethylene wear, ligament tension, and implant longevity [6,7,8,9,10,11,12]. In this context, the present data are clinically relevant primarily as an early radiographic accuracy signal for an imageless robotic platform in medial UKA. Absence of a statistically significant difference between robot-validated and postoperative radiographic values should not be interpreted as definitive proof of agreement; rather, the most informative findings are the low absolute deviations and low outlier rates observed descriptively in the present series.

The radiographic accuracy observed in the present study is consistent with the growing body of literature suggesting that robotic-assisted UKA improves implant positioning and reduces alignment outliers compared with conventional instrumentation [13,14,15,16,31,32,33,34,35]. Foissey et al. reported better implant positioning accuracy and improved implant survival in imageless robotic-assisted medial UKA compared with conventional implantation [13]. Avram et al. showed that robotic assistance may better restore the native joint line and reduce alignment outliers [14], while Kumar et al. and Lau et al. likewise reported improved restoration of target alignment and reduced outlier rates with robotic-assisted UKA [15,16]. In parallel, the systematic review by Robinson et al. and the meta-analyses by Zhang et al. and Bensa et al. supported better radiographic precision and fewer alignment outliers with robotic-assisted UKA than with conventional techniques [31,32,33]. Comparative and longer-term reports from other robotic platforms have also suggested favorable functional and survivorship outcomes [34,35,36,37,38,39,40]. Within this framework, the present series adds early data specifically for the ROSA^®^ Partial Knee platform, for which the currently available literature remains limited.

An additional finding of interest is that both coronal and sagittal tibial targets were reproduced with low observed deviation. This aspect is particularly relevant because sagittal accuracy, especially tibial slope control, remains one of the technically sensitive aspects of knee arthroplasty [8,11]. In the present series, planned, robot-validated, and postoperative radiographic tibial slope values remained close, and sagittal outlier rates were low. These data suggest that the imageless robotic workflow used in medial UKA may support accurate execution not only in the coronal plane but also in the sagittal plane.

Perioperative findings were favorable, with limited hemoglobin drop and relatively short operative times. These observations should nevertheless be interpreted within the design of the study. Because no control group was included, the present investigation does not allow conclusions regarding superiority in efficiency over conventional UKA or over other robotic systems. Similarly, the short-term clinical improvements observed in VAS, KSS, ROM, and UCLA activity score should be viewed as preliminary supportive findings rather than as definitive evidence of comparative clinical benefit, particularly because they were derived from outcome-specific paired subsets rather than from a fully mature uniform follow-up cohort. In this regard, the present study should not be interpreted as a mature PROM or survivorship investigation; its principal contribution remains the radiographic accuracy analysis.

The focus on medial UKA represents another relevant aspect of the present study. Medial and lateral UKA differ in indication, biomechanics, and technical demands, and restricting the analysis to medial procedures improved cohort homogeneity. Although robotic literature increasingly includes both compartments [38,40], the strongest body of evidence still concerns medial UKA. This makes the present findings more internally consistent and easier to interpret in relation to tibial coronal alignment and tibial slope targets.

The present findings should therefore be interpreted as early single-center evidence describing the radiographic accuracy of a newer imageless robotic platform in medial UKA. The current dataset is not intended to establish superiority over more mature technologies or definitive comparative clinical effectiveness. Rather, it documents that, in a standardized high-volume early-adopter setting, a recently introduced imageless system can reproduce planned tibial targets with low observed deviation on postoperative radiographs.

Limitations should nevertheless be acknowledged. First, the sample size was relatively small. However, this should be interpreted in light of the very recent introduction of the platform and the fact that the present study reflects an early consecutive experience in a high-volume early-adopter setting. Second, the study was conducted at a single center and all procedures were performed by a single experienced surgeon. This limits broader external generalizability, although it also enhances internal consistency by reducing procedural heterogeneity during an early technology evaluation. Third, no control group was included, so comparative conclusions cannot be drawn. Fourth, follow-up was short, and paired clinical follow-up was incomplete and outcome-specific, which limits interpretation of the exploratory clinical findings. Fifth, postoperative radiographic measurements were obtained from plain radiographs under routine clinical practice conditions around the first standardized follow-up visit. Although intraobserver reliability was good to excellent for the main parameters, radiographic measurements remain inherently subject to some variability. Finally, no formal interobserver reliability analysis was performed. These limitations indicate that the current study should primarily be regarded as an early radiographic accuracy study with secondary exploratory clinical observations.

The present study also has relevant strengths. All procedures were performed in a single institution using the same imageless robotic system, the same implant design, the same mid-vastus approach, and a standardized perioperative pathway. The cohort was homogeneous, restricted to medial UKA, and all cases had complete radiographic data for the primary analysis. In addition, intraobserver reliability for the main radiographic parameters was good to excellent, supporting internal consistency of the measurements. Taken together, these characteristics make the present series a meaningful early contribution to the still limited literature on ROSA^®^-assisted medial UKA.

## 5. Conclusions

In this preliminary single-center observational series, the ROSA^®^ Partial Knee System showed high early radiographic accuracy in reproducing planned tibial coronal alignment and tibial slope during medial UKA. Short-term clinical findings were encouraging but should be interpreted as exploratory because of incomplete paired follow-up. Larger comparative studies with longer follow-up are needed to determine the clinical relevance of this technical accuracy.

## Figures and Tables

**Table 1:** Radiographic parameters considered in the present medial UKA accuracy study. The table summarizes the imaging modality, interpretation, and minimum reporting requirements for each accuracy endpoint used in the manuscript.

Parameter	Imaging	Target/Interpretation	Minimum Data to Report
HKA	Full-length standing AP lower-limb radiograph	Residual mild varus is usually expected in medial UKA; overcorrection should be avoided	Pre-op mean ± SD, robot final value, post-op mean ± SD, paired n, *p*-value
Tibial coronal alignment (MPTA/tibial component coronal angle)	Standing AP knee or full-length AP depending on protocol	Should reproduce the planned under-corrected tibial target	Planned, robot-validated, post-op radiographic value, absolute deviation, outliers
Tibial slope	True lateral radiograph	Should reproduce the planned sagittal target without large outliers	Planned, robot-validated, post-op radiographic value, absolute deviation, outliers

**Table 2:** Demographic characteristics and perioperative data. Continuous variables are reported as mean ± standard deviation and range, while categorical variables are reported as absolute frequencies and percentages.

Variable	Mean ± SD/n (%)	Range
Patients, n	23	n.a.
Sex, male/female	14/9	n.a.
Age, years	68.4 ± 8.5	52–83
BMI, kg/m^2^	28.2 ± 4.1	23.5–37.9
Kellgren–Lawrence grade 3	9 (39.1%)	n.a.
Kellgren–Lawrence grade 4	14 (60.9%)	n.a.
Preoperative HKA (varus), °	5.4 ± 3.4	0.8–14.2
Preoperative hemoglobin, g/dL	14.0 ± 1.1	12.1–16.2
Hemoglobin drop, g/dL	0.8 ± 0.4	0.2–1.6
Robotic time, min	15.8 ± 2.7	11–22
Total surgical duration, min	29.4 ± 4.8	22–39

**Table 3:** Alignment outliers. Mean absolute deviation from target and the proportion of cases within 2° and 3° are reported for HKA, tibial coronal alignment (MPTA), and tibial slope.

Parameter	Mean Absolute Deviation, °	Within 2°, n (%)	Within 3°, n (%)
HKA	0.42 ± 0.38	21/23 (91.3%)	23/23 (100%)
Tibial coronal alignment (MPTA)	0.34 ± 0.29	22/23 (95.7%)	23/23 (100%)
Tibial slope	0.46 ± 0.39	21/23 (91.3%)	23/23 (100%)

**Table 4:** Comparison between robot-validated and achieved coronal values. Final robot-validated and postoperative radiographic coronal alignment values are reported for HKA and MPTA.

Parameter	Mean ± SD Robot	Mean ± SD Post-op Rx	*p*-Value
Final HKA, °	Varus 2.3 ± 0.7	Varus 2.6 ± 0.9	0.08
Tibial coronal alignment (MPTA), °	2.0 ± 0.5	2.1 ± 0.7	0.41

**Table 5:** Absolute difference between planned and achieved values. Mean absolute deviation, 95% confidence interval, and range are reported for HKA, MPTA, and tibial slope.

Parameter	Mean ± SD (95% CI)	Min–Max
HKA, °	0.42 ± 0.38 (0.26; 0.58)	0.0–1.5
Tibial coronal alignment (MPTA), °	0.34 ± 0.29 (0.21; 0.47)	0.0–1.1
Tibial slope, °	0.46 ± 0.39 (0.29; 0.63)	0.0–1.7

**Table 6:** Comparison between planned and achieved sagittal values. Planned, robot-validated, and postoperative radiographic tibial slope values are reported together with the *p*-value of paired comparison.

Parameter	Mean ± SD Planned	Mean ± SD Robot	Mean ± SD Post-op Rx	*p*-Value
Tibial slope, °	4.5 ± 0.6	4.6 ± 0.6	4.8 ± 0.8	0.27

**Table 7:** Early clinical outcomes at 3 months. Preoperative and 3-month postoperative values are reported together with the number of paired cases available for each analysis and the corresponding *p*-value.

Outcome	Preoperative	3-Month Follow-Up	Paired n	*p*-Value
VAS	8.1 ± 0.7	1.0 ± 0.9	14	<0.001
KSS Function	39.0 ± 6.2	86.5 ± 7.8	14	<0.001
KSS Knee	41.2 ± 7.0	91.8 ± 6.5	14	<0.001
ROM extension deficit, °	2.1 ± 3.4	0.2 ± 0.8	12	0.04
ROM flexion, °	121.5 ± 8.8	129.4 ± 6.7	12	0.01
UCLA activity score	3.2 ± 0.6	5.6 ± 1.0	9	0.008

**Footnote:** Paired analyses were performed only in patients with data available at both time points. Paired sample size differed across outcomes according to follow-up availability. No imputation of missing data was performed.

## Data Availability

The data presented in this study are available on request from the corresponding author. The data are not publicly available due to privacy and ethical restrictions.

## Supplementary Materials

The following supporting information can be downloaded at: https://www.mdpi.com/article/10.3390/jcm15103566/s1, Supplementary Material S1. STROBE Checklist; Supplementary Material S2. Study Flow Diagram.

## Abbreviations

UKA	unicompartmental knee arthroplasty
BMI	body mass index
HKA	hip–knee–ankle angle
MPTA	medial proximal tibial angle
VAS	visual analogue scale
KSS	Knee Society Score
(UCLA) activity score	University of California Los Angeles
FJS-12	Forgotten Joint Score-12
ROM	range of motion
OA	osteoarthritis
PROMs	patient-reported outcome measures
