# Supplementary material for: Accuracy of ROSA® Partial Knee System in Tibial Alignment During Medial Unicompartmental Knee Arthroplasty: An Observational Study
Source: J Clin Med. 2026 May 7;15(10):3566. doi: 10.3390/jcm15103566 (PMC13207302; doi:10.3390/jcm15103566)
Supplement: Supplementary file 1 [file jcm-15-03566-s001.zip › S1-Supplementary_STROBE_Checklist.pdf]

Supplementary Material S1. STROBE Checklist

Checklist of items that should be included in reports of cohort studies,
adapted to the present retrospective observational study on robotic-assisted medial UKA.

Section/Topic	Item No.	STROBE Recommendation	Location in Manuscript
**Title and abstract**	1	(a) Indicate the study's design with a commonly used term in the title or the abstract<br>(b) Provide in the abstract an informative and balanced summary of what was done and what was found	Title; Abstract
**Introduction**	2	Explain the scientific background and rationale for the investigation being reported	Introduction, paragraphs 1–5
	3	State specific objectives, including any prespecified hypotheses	Introduction, final paragraph
**Methods**	4	Present key elements of study design early in the paper	Materials and Methods, paragraph 1
	5	Describe the setting, locations, and relevant dates, including periods of recruitment, exposure, follow-up, and data collection	Materials and Methods, paragraph 2
	6	(a) Give the eligibility criteria, and the sources and methods of selection of participants<br>(b) For matched studies, give matching criteria and number of exposed and unexposed	Materials and Methods, paragraph 3; not a matched study
	7	Clearly define all outcomes, exposures, predictors, potential confounders, and effect modifiers. Give diagnostic criteria, if applicable	Outcomes of Interest
	8*	For each variable of interest, give sources of data and details of methods of assessment (measurement). Describe comparability of assessment methods if there is more than one group	Outcomes of Interest; Tables 1–7
	9	Describe any efforts to address potential sources of bias	Methods: paired analyses restricted to available data; standardized radiographic measurements by two observers; Discussion limitations
	10	Explain how the study size was arrived at	Consecutive series of all eligible cases during the study period; no formal sample-size calculation
	11	Explain how quantitative variables were handled in the analyses. If applicable, describe which groupings were chosen and why	Data Synthesis; Results; Tables 2–7
	12	(a) Describe all statistical methods, including those used to control for confounding<br>(b) Describe any methods used to examine subgroups and interactions<br>(c) Explain how missing data were addressed<br>(d) If applicable, explain how loss to follow-up was addressed<br>(e) Describe any sensitivity analyses	Data Synthesis; paired analyses restricted to available measurements; no multivariable adjustment; no sensitivity analyses
**Results**	13*	(a) Report numbers of individuals at each stage of study—e.g., potentially eligible, examined for eligibility, confirmed eligible, included in the study, completing follow-up, and analyzed	Results 6.1–6.4; Supplementary Flow Diagram

		(b) Give reasons for non-participation at each stage<br>(c) Consider use of a flow diagram	
	14*	(a) Give characteristics of study participants and information on exposures and potential confounders<br>(b) Indicate number of participants with missing data for each variable of interest<br>(c) Summarise follow-up time	Results 6.1 and 6.4; Tables 2 and 7
	15*	Report numbers of outcome events or summary measures over time	Results 6.2–6.4; Tables 3–7
	16	(a) Give unadjusted estimates and, if applicable, confounder-adjusted estimates and their precision<br>(b) Report category boundaries when continuous variables were categorized<br>(c) If relevant, consider translating estimates of relative risk into absolute risk	Results 6.2–6.4; Tables 3–7; no adjusted analyses
	17	Report other analyses done—e.g., analyses of subgroups and interactions, and sensitivity analyses	No prespecified subgroup or sensitivity analyses
**Discussion**	18	Summarise key results with reference to study objectives	Discussion, first paragraph
	19	Discuss limitations of the study, taking into account sources of potential bias or imprecision	Discussion, penultimate paragraph
	20	Give a cautious overall interpretation of results considering objectives, limitations, multiplicity of analyses, results from similar studies, and other relevant evidence	Discussion
	21	Discuss the generalisability (external validity) of the study results	Discussion, limitations/strengths paragraphs
**Other information**	22	Give the source of funding and the role of the funders for the present study and, if applicable, for the original study on which the present article is based	Title Page / Funding statement

**Items may include information that is presented in the flow diagram, tables, or supplementary material.*
