# Supplementary material for: Accuracy of ROSA® Partial Knee System in Tibial Alignment During Medial Unicompartmental Knee Arthroplasty: An Observational Study
Source: J Clin Med. 2026 May 7;15(10):3566. doi: 10.3390/jcm15103566 (PMC13207302; doi:10.3390/jcm15103566)
Supplement: Supplementary file 1 [file jcm-15-03566-s001.zip › S2-Supplementary_Flow_Diagram.pdf]

Supplementary Material S2. Study Flow Diagram

Patient flow for the retrospective cohort of robotic-assisted medial unicompartmental knee arthroplasty.

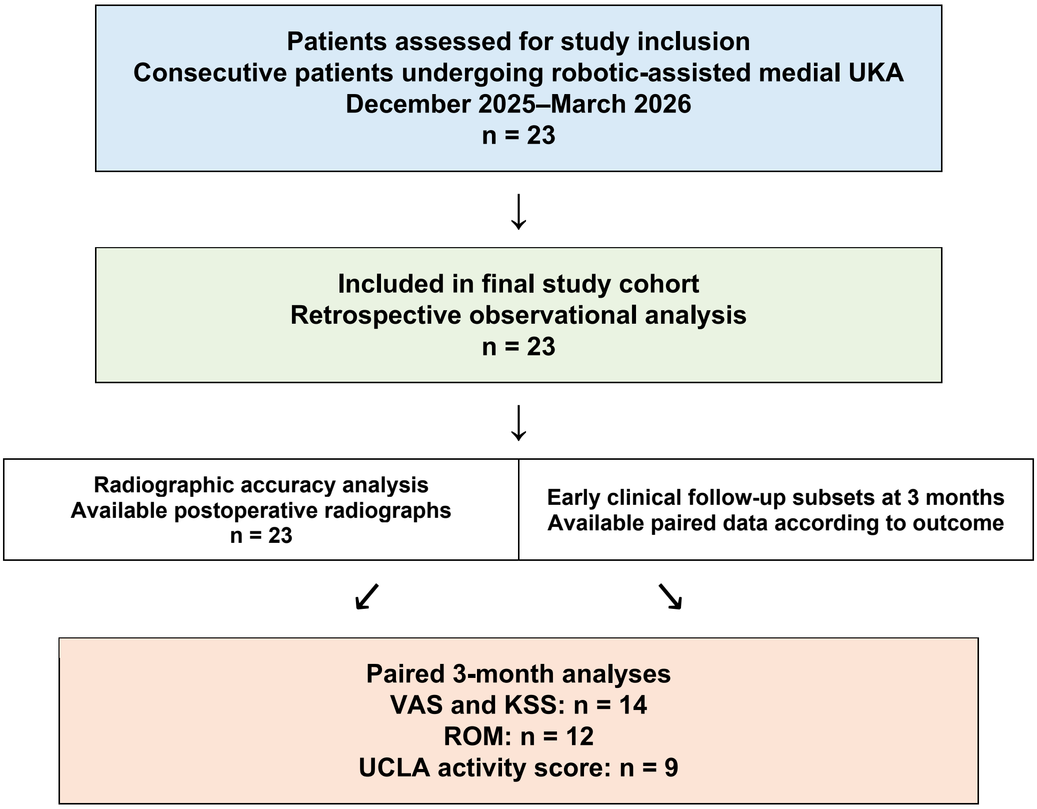

Abbreviations: UKA, unicompartmental knee arthroplasty; VAS, visual analogue scale; KSS, Knee Society Score; ROM, range of motion; UCLA, University of California Los Angeles activity score.
